# Gut yeasts accelerate chill coma recovery in *Drosophila melanogaster*

**DOI:** 10.1242/jeb.251533

**Published:** 2026-05-05

**Authors:** Yanira Jiménez-Padilla, Brent J. Sinclair

**Affiliations:** ^1^ Department of Biology, University of Western Ontario, London, Ontario N6A 5B7, Canada; ^2^ Department of Entomology, Cornell University, Ithaca, NY 14853, USA

**Keywords:** Cold, Gut microbiota, Insect symbionts, *Lachancea kluyveri*, Thermal biology, Yeasts

## Abstract

The role of microbial symbionts in host stress tolerance remains underexplored. Gut microbiome studies in *Drosophila melanogaster* have largely focused on bacteria, whereas yeasts have been assumed to provide nutrition rather than engage in true symbiosis. We explored the effect of gut yeasts on chill coma recovery time (CCRT, a proxy for cold tolerance) and its yeast species-specificity and dependence on live yeast cells. We generated flies with distinct gut microbiota conditions: axenic (microbe-free), with their native microbiota (derived from the microbes associated with flies conventionally reared in our colony), or gnotobiotic flies mono-associated with either live or heat-killed yeasts (*Saccharomyces cerevisiae*, not normally associated with *Drosophila* guts, and three species previously isolated from wild flies: *Lachancea kluyveri*, *Pichia kluyveri* and *P. nakasei*). We quantified yeast abundance, sex differences in yeast ingestion, and measured CCRT after exposure to 0°C for 8 h. Female axenic flies recovered 42% more slowly than those with their native microbiota, but this delay was fully rescued by live *L. kluyveri*, *P. kluyveri* or *P. nakasei*, not by *S. cerevisiae* or dead yeasts. The effect was rapid (occurred within 48 h), sex-specific (restricted to females) and appeared to be dose-dependent. We also confirmed that yeasts in the gut are alive, facilitating a true (albeit transient) symbiotic interaction. Our findings show that yeast symbionts may contribute to natural variation in thermal tolerance and may broadly impact host phenotypes. Excluding yeasts or assuming a solely nutritional role risks overlooking key symbiotic interactions that have profound functional consequences.

## Teaser

**Highlighted Article:** Live yeasts enhance cold tolerance in *Drosophila melanogaster*, with rapid, yeast species-specific and host sex-dependent effects, indicating a symbiotic role for gut yeasts in insects beyond nutrition.

## INTRODUCTION

Most animals form symbioses with microbes that inhabit their external and internal epithelia and sometimes specialised cells (Douglas, 2022), affecting host immunity, development, digestion, reproduction, longevity and behaviour (Douglas, 2022). For ectotherms, temperatures outside of their optimal range limit their activity, growth, reproduction and geographic distribution (Angilletta, 2009). Seasonal changes in temperature modify the microbiota of temperate insects (e.g. Ferguson et al., 2018; Hou et al., 2021). In turn, microbial symbionts influence their hosts' thermal biology. For example, *Rickettsia*-hosting *Bemisia tabaci* whiteflies tolerate higher temperatures than their *Rickettsia*-free counterparts (Brumin et al., 2011).

Most microbial symbionts are in the gut of the host; they include bacteria, fungi, archaea, protists and viruses (Douglas, 2022). Perturbing gut microbes with antibiotics reduces thermal tolerance in green frog tadpoles (Fontaine et al., 2022), and microbiota-deplete *D. melanogaster* have reduced cold tolerance (Henry and Colinet, 2018). Conversely, heat exposure modulates gut microbiome diversity in a desert lizard (Yang et al., 2024). Because antibiotics perturb the microbiota only haphazardly and comparing sterile animals with their conventionally reared counterparts cannot distinguish the contributions of specific microbes, studies seldom yield causal connections between the gut microbiota and host thermal biology (Ridley et al., 2013). Furthermore, 16S rRNA metagenomics characterises only bacteria, omitting information about other components of the microbiota, such as fungi (Forbes et al., 2019).

Ectotherm thermal tolerances are phenotypically plastic and geographically variable (Angilletta, 2009). Because the gut microbiota is environmentally responsive (Ferguson et al., 2018; Petersen et al., 2023) and varies among populations (Wong et al., 2013), it could contribute to this phenotypic plasticity. This means that locally attuned gut microbes could facilitate success in novel environments, including for invasive species (Li et al., 2022; Petersen et al., 2023; Taerum et al., 2013). The generality of these relationships remains unproven; for example, although low temperature rearing changes the *D. melanogaster* gut microbiota, introducing these cold-adapted microbes to control flies does not affect their cold tolerance (Moghadam et al., 2018).

Yeasts are ubiquitous in animal gut microbiota (Parle, 1957; Stefanini, 2018) as both opportunistic pathogens and beneficial symbionts (Bilal et al., 2023; Gatesoupe, 2007). Gut yeasts influence host digestion (e.g. in ruminants and termites; Schäfer et al., 1996; Suntara et al., 2021), nutrient provisioning (e.g. in beetles and drosophilids; Davis, 2015; Starmer and Aberdeen, 1990), immune defence (e.g. in honey bees; Tauber et al., 2019) and development (e.g. in drosophilids; Jiménez-Padilla et al., 2024), but their role in host thermal performance is little studied. In fish, pig and dairy farming, live *S. cerevisiae* is used as a nutritional supplement because it reduces heat stress and improves productivity (Du et al., 2022; Labussière et al., 2022; Ran et al., 2015). Live yeast was shown to increase *D. melanogaster* cold tolerance (Colinet and Renault, 2014), but again *S. cerevisiae* was added as a nutritional supplement rather than a potential symbiont.

*Drosophila* acquire their gut microbiota from their surroundings. Geography and habitat therefore determine microbial community composition more than the host *Drosophila* species (Bakula, 1969; Lachance et al., 1995). Yeasts (mainly in the genera *Hanseniaspora*, *Pichia* and *Saccharomyces*) are a major component of the gut microbiota of *Drosophila* flies (Chandler et al., 2012). The relative simplicity of gut yeast communities and the ability to rear axenic flies (without microbes) or flies with known gut microbe compositions (i.e. gnotobiotic) makes for a tractable system for exploring yeast–host interactions (e.g. Jiménez-Padilla et al., 2024).

Insect geographic distributions are constrained (at least in part) by their low temperature tolerance (Kellermann et al., 2012). At their critical thermal minimum, insects enter chill coma; the time to regain coordinated movement after rewarming (chill coma recovery time, CCRT) is an integrative proxy for cold tolerance in *Drosophila* and other insect species (Overgaard and MacMillan, 2017). CCRT varies among species and populations. For example, temperate *Drosophila* species have faster CCRT than relatives from tropical places (Andersen et al., 2015; Gibert et al., 2001) and warm-climate populations of *Drosophila* spp. have slower CCRT than their temperate counterparts (Sinclair et al., 2015). However, both CCRT and gut microbiota composition are plastic in response to temperature and diet (Andersen et al., 2013; Colman et al., 2012; Moghadam et al., 2018; Ransberry et al., 2011). It is unclear whether the changes in microbiota play a role in coincident changes in CCRT.

Here, we investigated the influence of gut yeasts on the cold tolerance of *D. melanogaster*. We show that the *Drosophila*-associated yeast *Lachancea kluyveri* persists in the gut longer than *Saccharomyces cerevisiae* (which is used in lab diets, but not commonly found in *Drosophila* in nature). Live (but not dead) gut yeasts impact CCRT in a yeast species- and host sex-specific manner: live *L. kluyveri*, but not *S. cerevisiae*, reduced female recovery time, and neither species affected males (which consume much less yeast than females). We show that these relationships hold across multiple populations of *D. melanogaster* and several different *Drosophila*-associated yeasts, suggesting a general and substantial impact of gut yeasts on *Drosophila* thermal biology.

## MATERIALS AND METHODS

### Experimental animals

For most of our experiments, we used a population of outbred *Drosophila melanogaster* Meigen 1830 collected from London, Ontario, Canada (43.00°N, 81.25°W), in 2008 (Marshall and Sinclair, 2010), treated with tetracycline in 2012 to remove *Wolbachia* (Salehipour-Shirazi et al., 2017). To compare fly populations, we acquired *D. melanogaster* from two locations in Australia [tropical: Innisfail, Queensland (17.53°S, 146.03°E); temperate: Melbourne, Victoria (37.73°S, 145.45°E)] collected in 2016 by Dr Carla Sgró, Monash University. We eliminated *Wolbachia* from these populations with tetracycline (Hoffmann, 1988). We reared flies at 21.5±1°C and 60±5% relative humidity, under a 13 h:11 h light:dark photoperiod.

We maintained outbred populations as described by Jiménez-Padilla et al. (2024), mating ca. 750 adult flies in 3.8 l plastic cages and distributing eggs at low density (ca. 50 eggs per vial) in 35 ml vials containing 10 ml of Tucson food (1 liter dH_2_O, 15 g dry active yeast, 43 g sugar, 27 g cornmeal, 10 g agar, 4 ml propionic acid; Markow and O'Grady, 2006).

### Yeast cultures

*Saccharomyces cerevisiae* (strain UWOPS 92-222.2), *Lachancea kluyveri* (strain NRRL.Y-212651), *Pichia kluyveri* (strain UWOPS 91.603.2) and *Pichia nakasei* (strain UWOPS 91.641.9) came from the Department of Biology, University of Western Ontario yeast collection. For microscopy, we used green fluorescent protein (GFP)-expressing *S. cerevisiae* (W303), provided by Dr P. Lajoie, University of Western Ontario. These species were selected to encompass both *S. cerevisiae* (standard in laboratory *Drosophila* rearing) and yeasts more typically associated with *Drosophila* in natural environments, including *P. kluyveri*, which is commonly recovered from wild *D. melanogaster* (Chandler et al., 2012; Lachance et al., 1995). We cultured all species for experiments at 25°C and maintained working cultures at 15°C, renewing them from stocks stored at −80°C every 2–3 months. *Saccharomyces cerevisiae*, *L. kluyveri* and *P. kluyveri* prefer warmer temperatures, whereas psychrophilic *P. nakasei* can also grow at 4°C. We cultured yeasts on yeast–malt agar (YM agar: 1% w/v glucose, 0.5% peptone, 0.3% malt extract, 0.3% yeast extract, 2% agar). We prepared yeasts for experiments by plating material from three working culture colonies on YM agar and incubating (3 days, 25°C), suspending the resulting colonies in sterile phosphate-buffered saline (PBS, P4417, Millipore-Sigma) and calculating concentration using absorbance at 560 nm (calibrated to standard curves). To heat-kill yeasts, we exposed suspensions to 60°C for 30 min (Jiménez-Padilla et al., 2024).

### Rearing axenic and gnotobiotic flies

We transferred ca. 200 adult flies (5–10 days old) to oviposition cages (diameter=3.5 cm, height=5.8 cm) capped with a Petri dish of apple juice agar (100 ml juice, 100 ml dH_2_O, 4 g agar) topped with a paste of commercially sourced inactive *S. cerevisiae* and distilled water. On day 4, we replaced the Petri dish with a new apple agar dish without yeast paste and allowed the females to oviposit for 3 to 5 h.

We reared axenic flies according to Jiménez-Padilla et al. (2024). Briefly, we transferred ca. 30 eggs to an autoclaved nylon filter (20 µm pore, diameter=24 mm, NY2002500, Millipore-Sigma, Oakville, ON, Canada), submerged them in 70% ethanol (5 min), and rinsed them (3×) with sterile PBS. We inverted the filter onto a thin layer of sterile yeast–sugar agar (1.5 g agar, 1.5 g active yeast, 4.3 g table sugar, 100 ml dH_2_O) to release the eggs and placed a square of agar with eggs into a vial containing autoclaved Tucson food (without propionic acid to avoid inhibiting microbial growth). We plugged these vials with sterile cellulose acetate plugs and paper covers to prevent contamination and reared flies under standard growth conditions until adults emerged.

When transferring flies to treatments, we spot-plated three male and three female homogenised flies and a ca. 3 μl sample of food on YM agar and incubated at 25°C to confirm axenic state (we discarded any vials that showed unexpected microbial growth after 3 or 7 days). We prepared 13 vials per treatment to allow us to remove any that were contaminated. Where there was no contamination, we randomly selected 10 vials per treatment, discarding the remaining vials to maintain a balanced design.

### Treatments

**Fig. 1.**
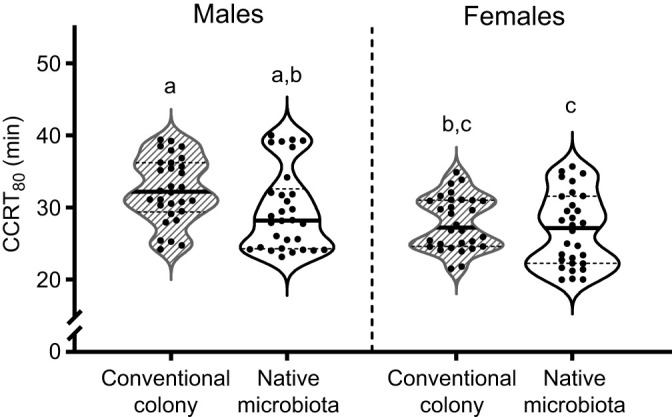
**The chill coma recovery time (CCRT) of *Drosophila melanogaster* flies in the native microbiota group did not differ from that of the conventionally reared flies.** We collected the eggs for both groups from the same parents and at the same time. The eggs for the native microbiota were surface sterilised in ethanol and placed in food inoculated with fly homogenates to reintroduce their normal microbes. The eggs for the conventional colony group were transferred directly to standard Tucson food without further manipulation. We compared groups with two-way ANOVA (treatment: *F*_1,116_=1.84, *P*=0.178; sex: *F*_1,116_=11.53, *P*<<0.001; interaction: *F*_1,116_=0.05, *P*=0.819); different letters indicate significant differences (Tukey's HSD, *P*<0.05). Each point represents the CCRT_80_ from a group of five flies from a single vial (*n*=30 vials per treatment, three cohorts pooled).

We transferred adult axenic flies (48 h post-eclosion) under light CO_2_ anaesthesia to vials randomly assigned to treatments (Table 1) and allowed them to eat for 48 h before measuring CCRT. Flies were not sex-separated prior to transfer and, therefore, were assumed to have mated. For the native microbiota group, we introduced microbes from the conventional colony to axenic eggs; CCRT did not differ between conventionally reared and native microbiota flies (Fig. 1). Yeast suspensions were prepared as above, and we used eight times the concentration of live yeast for the dead yeast treatments to account for live yeast proliferation during the experiment. In previous work (Jiménez-Padilla et al., 2024), we found that the effects of heat-killed yeasts on *Drosophila* development time did not increase beyond this concentration. Vials were labelled with random codes to blind individuals recording CCRT to the treatment groups. We spot-plated all treatment suspensions on YM agar and incubated them (25°C, 3 days). If any treatment had unexpected microbial growth, the experiment was repeated and all vials were discarded.

**Table 1.** Treatments to examine the impact of yeasts on *Drosophila* chill coma recovery time

Treatment group	Treatment suspension
Axenic	Sterile PBS
Native microbiota	Three conventionally reared adult flies homogenised in 200 µl sterile PBS
Gnotobiotic	Live yeast suspended in sterile PBS at 1.3×10^8^ cells ml^−1^
Dead yeast	Yeast cells suspended in sterile PBS at 1.04×10^9^ cells ml^−1^ (8× the concentration of live yeast) and heat-killed by exposure to 60°C for 30 min

Sterile Tucson food vials were inoculated with 10 µl of a treatment suspension and flies allowed to feed for 48 h prior to measuring chill coma recovery time. PBS, phosphate-buffered saline.

### Yeast viability and persistence in the *D. melanogaster* gut

To determine whether yeasts survive in the gut of *D. melanogaster*, we transferred axenic flies 48 h post-eclosion to Tucson food vials inoculated with 10 µl of GFP-*S. cerevisiae* or *L. kluyveri* suspensions (1.3×10^8^ cells ml^−1^). After 24 h, we lightly anaesthetised the flies with CO_2_ and collected 3 flies per vial (5 vials per yeast). We surface-sterilised the flies in 70% ethanol (1 min), rinsed in sterile PBS and dissected their gut. We wet-mounted the gut and frass for observation under phase-contrast (100× objective lens with oil immersion), fluorescence (488 nm excitation) and differential interference contrast (DIC; Axio Imager Z1, Zeiss, Toronto, ON, Canada). We identified yeast cells and observed reproduction (budding and ascospore production) and viability (membrane integrity and refractiveness).

To determine how long yeasts remain in the adult gut, we reared axenic flies (50 eggs per vial) and transferred them 48 h post-eclosion to Tucson food vials inoculated with live *S. cerevisiae* or *L. kluyveri*. After 24 h (day 0) in these treatment vials (five vials per yeast species), we removed three males and three females under light CO_2_ anaesthesia for later dissection. We transferred the remaining flies to a sterile Tucson food vial (without added yeasts) and returned them to standard rearing conditions. We repeated these transfers every 24 h until no yeast growth was detected. To detect yeasts, we surface-sterilised flies, dissected and homogenised their guts in 100 µl sterile PBS, and spread-plated them on YM agar (diluted 100× for the first 2 days for females of both species and 10× on day 3 for *L. kluyveri*). We incubated these plates at 25°C and counted colony-forming units (CFU) after 48 h.

To compare the amount of yeast ingested by males and females, we placed ca. 30 axenic flies (48 h post-eclosion) into a sterile vial containing 10 ml of 2% agar for 30 min before transferring them to another sterile vial (nine groups of flies total) containing a 5×5 mm square of YM agar with three colonies of GFP-expressing *S. cerevisiae*. After 30 min, we then surface-sterilised two males and two females from each vial, dissected them and placed their guts on a microscope slide inside a ring of Carolina observation gel (132700, Carolina, Burlington, NC, USA). We conducted all dissections and gut inspections at the same time of day to control for potential circadian effects on feeding behaviour. We observed the crops of yeast-fed males and females, and food-deprived flies (kept in 2% agar vials) with fluorescence (488 nm excitation) and DIC microscopy. We also monitored the number of flies attracted to yeast paste (*L. kluyveri* dyed with Trypan Blue) and compared the amount of yeast ingested by male and female flies. We used abdomen distension and colouration as a proxy for volume consumed (Edgecomb et al., 1994; Fig. S2).

### Measuring chill coma recovery time

To measure CCRT, we placed the vials (10 vials per treatment) in ice-water slurry (0°C) for 5 min to immobilise the flies. We sorted them on a Petri dish maintained in contact with the ice-water slurry while working in a 4°C chamber and separated five males and five females per vial into single-sex 1.7 ml microcentrifuge tubes, sealed the tubes in a plastic bag and submerged them at 0°C for 8 h. After exposure, we transferred all the flies from each tube into a single well of a six-well tissue culture plate at room temperature (ca. 21°C) and recorded the time taken from being removed from the ice-water slurry to being able to stand for each individual (i.e. CCRT). When all flies had recovered, we cold-immobilised them again and haphazardly selected three flies from each well for spot-plating of a whole-body homogenate as described above. As above, we discarded any vials with unexpected microbial growth. We repeated each CCRT experiment with three cohorts (each derived from a different parental generation at least 3 weeks apart) for each fly population.

### Relationship between CCRT and gut yeast abundance

To determine whether recovery time is influenced by variation in the number of yeast cells housed by an individual fly, we transferred five vials of axenic flies (48 h post-eclosion) to sterile Tucson food vials inoculated with live *L. kluyveri* (1.3×10^8^ cells ml^−1^). We randomly selected three vials and measured CCRT (as above). We then surface-sterilised the flies, dissected and homogenised their guts in 50 μl PBS (as described above), and spread-plated a 10-fold dilution of the homogenate on YM agar. We counted CFU after 48 h of incubation at 25°C. We repeated this experiment for three different cohorts.

### Statistical analysis

We performed all analyses using GraphPad Prism (v10.1.2 for Windows, GraphPad Software, Boston, MA, USA) or R v4.4.1 (https://www.r-project.org/). Linear mixed-effects models were fitted in R using the packages *lme4* v1.1.38 (Bates et al., 2015) and *lmerTest* v3.2.0 (Kuznetsova et al., 2017). We compared treatment effects on recovery time using CCRT_80_, defined as the time at which 4 of 5 flies within a vial had recovered (i.e. the 80% recovery time; Jakobs et al., 2015). We first tested whether treatment effects differed among cohorts by fitting a model that included cohort and its interactions; no significant interactions involving cohort were detected. We then fit a linear mixed-effects model with cohort included as a random intercept [CCRT_80_∼Treatment×Sex+(1|Cohort)]. The estimated cohort variance was zero (singular fit), indicating negligible between-cohort variation, so we conducted subsequent analyses of CCRT_80_ with pooled cohorts using a two-way ANOVA with treatment and sex as fixed factors, followed by Tukey's *post hoc* tests.

We re-analysed our data using (equally valid) survival analyses, and present cumulative CCRT recovery curves for all individual flies for each sex, treatment and cohort in Figs S2 and S3 and Table S1. Because we conducted the experiments across three independent cohorts, we first tested for cohort effects within each treatment (within sex) using log-rank (Mantel–Cox) tests and detected no significant cohort differences; we therefore pooled cohorts in subsequent analyses. We compared treatments using a global log-rank test within each sex (d.f.=5). Survival analyses yielded conclusions wholly consistent with the CCRT_80_ mixed-model/ANOVA results, which we present in the Results to facilitate interpretation of inter-group comparisons.

We analysed the relationship between CCRT and the abundance of *L. kluyveri* (CFU per fly) using a linear mixed-effects model. CFU was included as a fixed effect, and vial was included as a random intercept to account for non-independence of flies within vials [CCRT∼CFU+(1|vial)]. Cohort was initially included as an additional random effect, but its variance was estimated as zero and was therefore removed from the final model. Degrees of freedom and *P*-values were estimated using Satterthwaite's approximation (lmerTest). Model assumptions were assessed by inspection of residuals.

## RESULTS

### Viability and persistence of yeasts in the gut of *D. melanogaster*

**Fig. 2.**
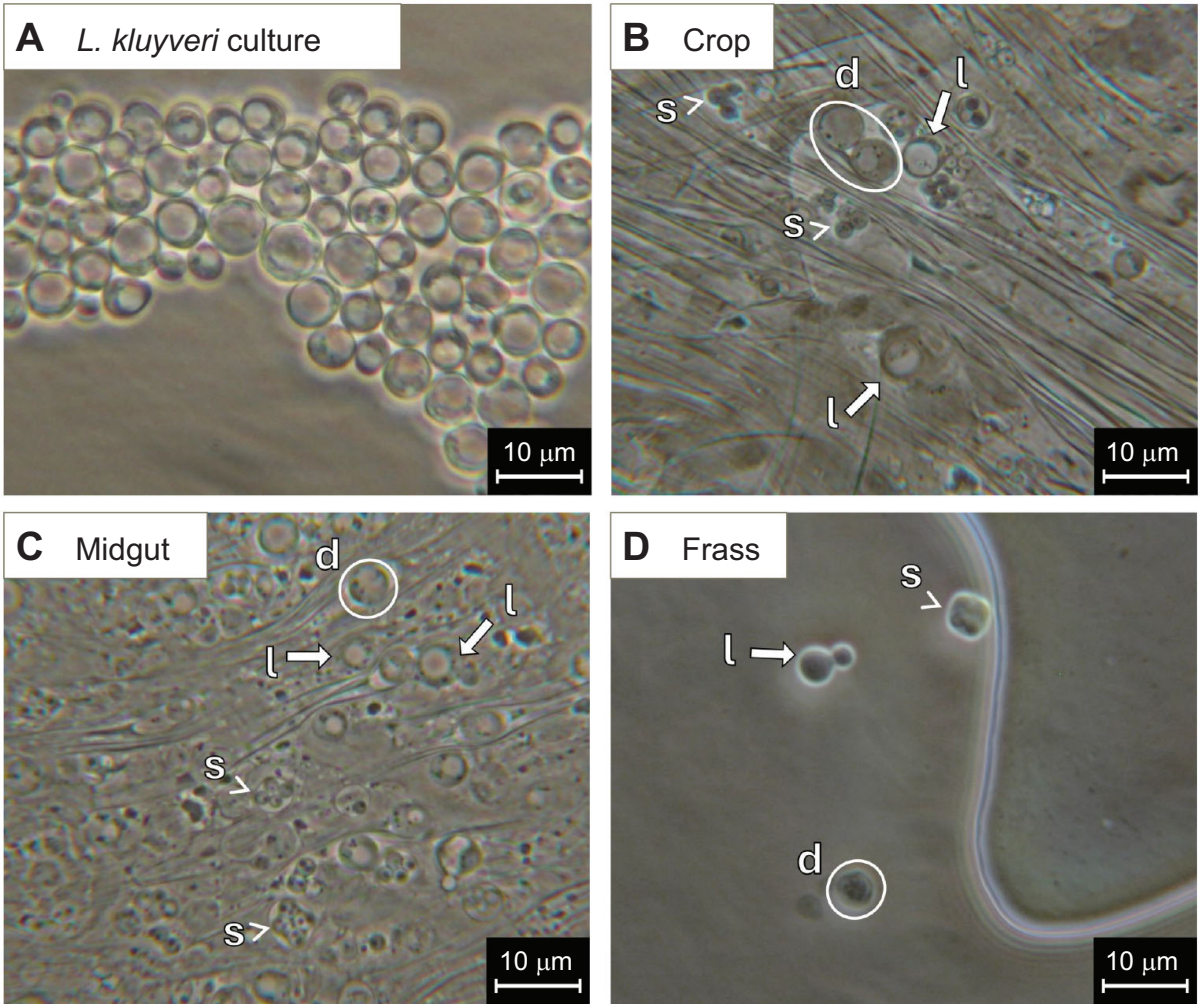
**Examples of *Lachancea kluyveri* cells recovered from the gut and frass of adult *D. melanogaster*.** (A) Live yeast cells on YM agar; (B) dissected crop (striations are muscle fibres); (C) posterior midgut; (D) yeast recovered from frass. d, digested cells; l, live cells; s, spores.

**Fig. 3.**
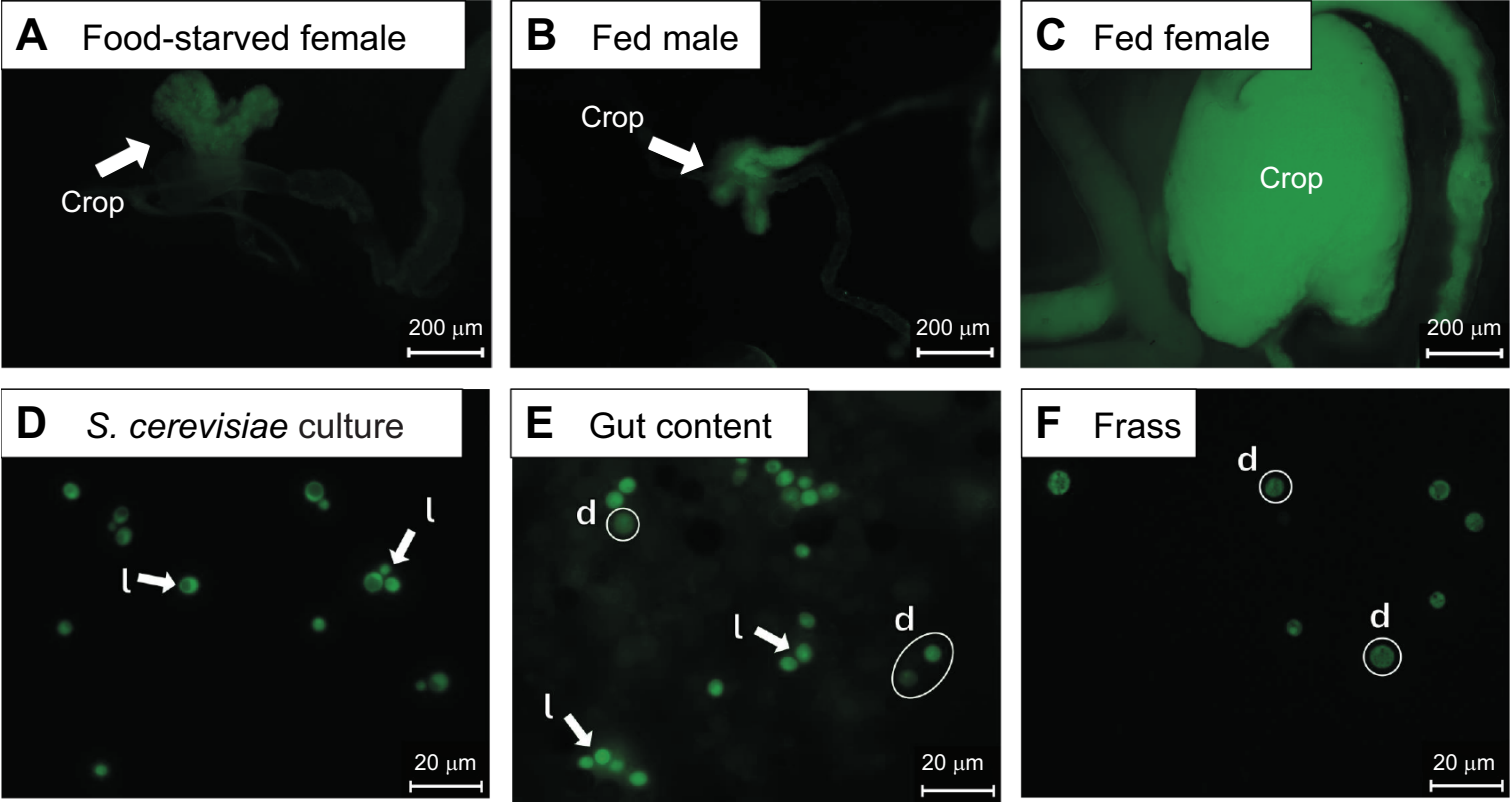
**Examples of *Drosophila melanogaster* crops and gut contents after ingestion of GFP-*Saccharomyces cerevisiae*.** (A) Crop from a food-deprived female. (B) Crop from a yeast-fed male showing some fluorescence due to yeast ingestion, but little distension. (C) Distended female crop after yeast consumption*.* Fluorescing GFP-*Saccharomyces cerevisiae* cultures in (D) YM agar, (E) female midgut and hindgut content, and (D) digested GFP-*S. cerevisiae* cells in the frass of female flies. d, dead yeast cells; l, live cells.

We observed live yeast cells from both GFP-*S. cerevisiae* and *L. kluyveri* species in fly guts 24 h after inoculation (Figs 2 and 3). Only *L. kluyveri* sporulated in the gut; the spores were recovered along the whole digestive tract and in freshly deposited frass (Fig. 2). *Saccharomyces cerevisiae* did not form spores, and the frass of *S. cerevisiae*-fed flies contained only digested yeast cells (spheroplasts, Fig. 3F), which form when the cell wall has failed and the cell is enclosed only by a membrane (Kelly and Nurse, 2011).

Female flies ingested more yeast than males. The crops of females fed with GFP-*S. cerevisiae* were markedly distended and exhibited strong fluorescence compared with food-deprived females and fed males (Fig. 3). Male flies also ingested yeast, but to a lesser extent. Male crops remained unexpanded, similar in size and shape to the crops of food-deprived females; therefore, we did not image (empty) guts from food-deprived males, which were indistinguishable from those of fed males (Fig. 3). We also observed more Trypan Blue-stained *L. kluyveri* in female gnotobiotic flies compared with their male counterparts (Fig. S1).

**Fig. 4.**
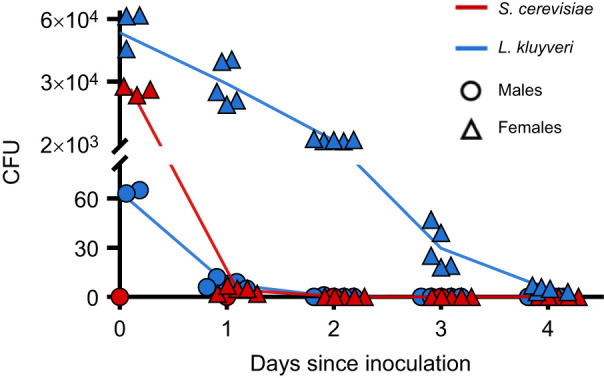
**Persistence of the yeasts *S. cerevisiae* and *L. kluyveri* in the *D. melanogaster* gut.** Each point represents the median calculated from three flies from a given vial. The lines are connected at the mean±s.e.m. of each vial (*n*=5 vials per treatment per sex). Flies were transferred daily to new sterile food vials until no more yeast was recovered.

The persistence of yeasts in the fly gut depended on both yeast species and fly sex. We recovered an average of 6.1×10^4^ CFU from the guts of female flies fed with live *L. kluyveri* for 24 h (day 0, Fig. 4). This number nearly halved (3.2×10^4^ CFU) 24 h after the first transfer to sterile food vials (day 1). By day 2, yeast abundance had dropped to ca. 6% (3.9×10^3^ CFU) of the initial amount recovered. By contrast, male flies from the same vials averaged only 68 CFU after feeding on *L. kluyveri* for 24 h, 8 CFU 24 h after the first transfer, and we did not recover any *L. kluyveri* from males after day 2 (Fig. 4). Female flies fed with live *S. cerevisiae* for 24 h began with around half the number of CFU (2.5×10^4^ CFU) compared with those fed on *L. kluyveri*, and we recovered fewer than ten *S. cerevisiae* CFU on day 2 and none thereafter. We recovered fewer than 5 CFU from the guts of *S. cerevisiae*-fed males on day 0 and none after transfer.

### Live *L. kluyveri* reduces CCRT in females but not males

**Fig. 5.**
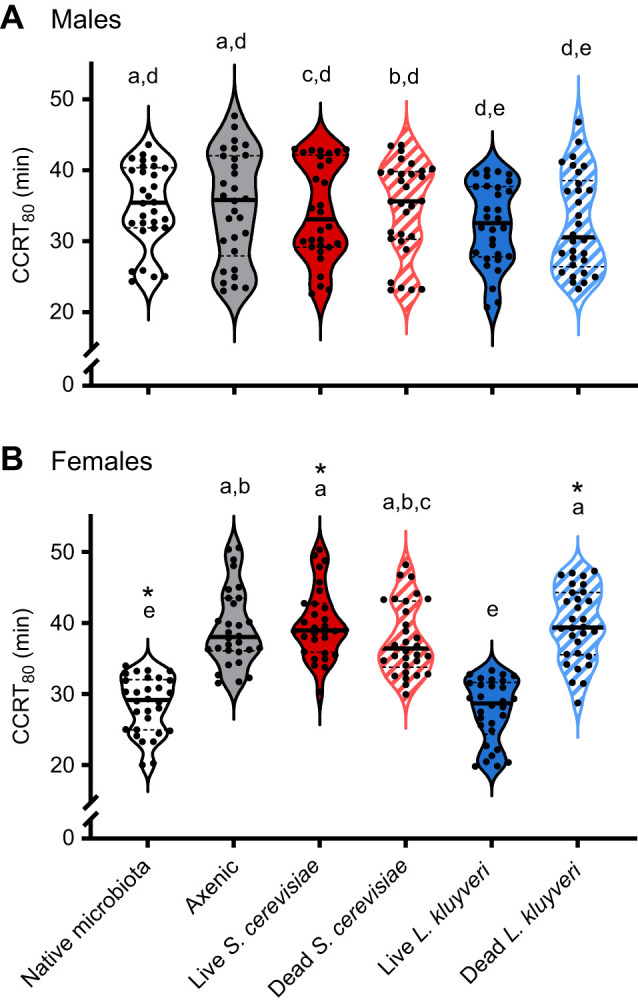
**Effect of gut yeasts on adult *D. melanogaster* CCRT.** CCRT_80_ is the recovery time of the fourth fly in a group of five. Males (A) and females (B) were included in each experimental run, and the analysis was conducted across the entire dataset, but are displayed separately for clarity. We compared groups with two-way ANOVA; different letters indicate significant among-group differences (Tukey's HSD, *P*<0.05), and asterisks indicate sex differences within the same treatment group. Each point represents the CCRT_80_ from a group of five flies from a single vial (*n*=30 vials per treatment, three cohorts pooled). CCRT data is also presented as Mantel–Cox curves in the Figs S2 and S3.

CCRT was influenced by yeast species and host sex, and only when the yeast was alive (treatment: *F*_5,348_=16.23, *P*<*<*0.001; sex: *F*_1,348_=6.38, *P*<*<*0.01; interaction: *F*_5,348_=14.4, *P*<*<*0.001; Fig. 5). Gut microbes (or their absence) did not affect CCRT in male flies (*P>*0.99; Fig. 5A). Axenic females had slower chill coma recovery relative to those with a native microbiota, and the CCRT_80_ of axenic females (39±5 min) did not differ from those hosting live *S. cerevisiae* (40±5 min; *P>*0.99; Fig. 5B). However, feeding females on live *L. kluyveri* (27±4 min) fully recovered the native microbiota CCRT_80_ phenotype of flies reared with their full gut microbial complement (28±4 min; *P>*0.99; Fig. 5B). Dead yeast, either *S. cerevisiae* or *L. kluyveri*, had no effect on the CCRT_80_ of male or female flies (Fig. 5).

**Fig. 6.**
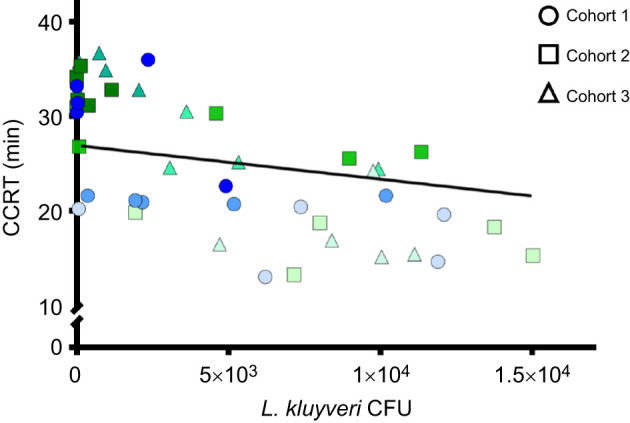
**Gut yeast (*L. kluyveri*) abundance determines CCRT.** Each data point represents a single female fly (*n*=45 total; 15 per cohort), pooled from three cohorts. Point shape denotes cohort, and colour shade distinguishes vials within cohorts. The solid line shows the fixed-effect estimate from a linear mixed-effects model including vial as a random intercept; CFU was a significant negative predictor of CCRT (β=−3.6×10^−4^, *P*=0.01).

The number of yeasts in the gut significantly influenced the CCRT of female flies (Fig. 6). CCRT decreased with increasing *L. kluyveri* abundance. In a linear mixed-effects model including vial as a random intercept, CFU was a significant negative predictor of CCRT (β=−3.6×10^−4^±1.3×10^−4^ s.e., *P*=0.01), corresponding to a reduction in recovery time of ca. 1 min per 2.8×10^3^ CFU. We observed within-vial variation in both CFU and CCRT (Fig. 6), but this variation is consistent with that observed in cumulative recovery curves (Fig. S2), indicating that we are capturing individual-level biological variability expected with outbred populations.

### Yeast species naturally associated with *Drosophila* reduce female CCRT

**Fig. 7.**
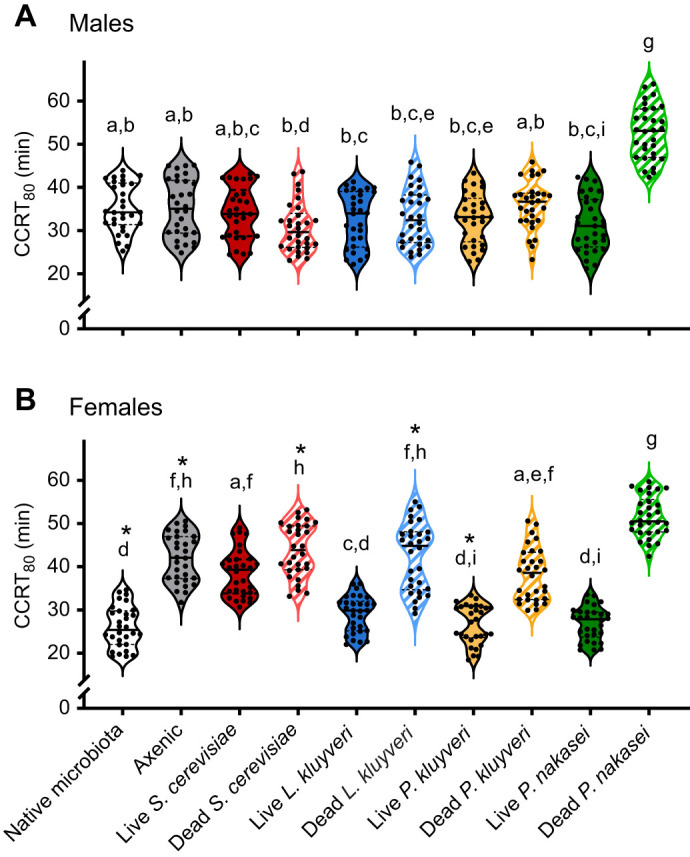
**Effect of four species of yeasts on *D. melanogaster* CCRT**. CCRT_80_ is the recovery time of the fourth fly in a group of five. Experiments and analyses were conducted simultaneously for both sexes, but they are separated here for clarity. We compared groups with two-way ANOVA; different letters indicate significant among-group differences (Tukey's HSD, *P*<0.05), and asterisks indicate sex differences within the same treatment group. Each point represents the CCRT_80_ from a group of five flies from a single vial (*n*=30 vials per treatment, three cohorts pooled).

Other yeast species isolated from wild-caught *Drosophila* spp. also influence adult CCRT depending on fly sex and whether the yeast was alive or dead in the same manner as we found for *L. kluyveri* (treatment: *F*_9,580_=82.46, *P*<*<*0.001; sex: *F*_1,580_=6.38, *P*<*<*0.02; interaction: *F*_9,580_=23.74, *P*<*<*0.001; Fig. 7). No yeast species affected male CCRT except for males fed on dead *P. nakasei*, which took 50% longer to recover (CCRT_80_=53±6 min) compared with axenic males (CCRT_80_=35±6 min, *P*<*<*0.001; Fig. 7A). Unlike the *S. cerevisiae*, which is not normally associated with *Drosophila* and does not affect CCRT_80_ (see also Fig. 5), yeast species originally isolated from *Drosophila* guts reduced female CCRT (*L. kluyveri*: CCRT_80_=29±4 min, *P. kluyveri*: CCRT_80_=27±5 min, and *P. nakasei*: CCRT_80_=27±4 min). Dead *L. kluyveri* and *P. kluyveri* did not change female CCRT compared with axenic flies (Fig. 7B), but dead *P. nakasei* increased female CCRT by 24% relative to their axenic counterparts (Fig. 7B).

Both male and female *D. melanogaster* from tropical Australia had inherently slower CCRT compared with their temperate Australian or Ontario counterparts (see native microbiota flies in Figs S4 and S5). Live yeasts influenced CCRT in these populations in the same manner as we describe for our Ontario-collected flies (Figs S4 and S5). *Saccharomyces cerevisiae* did not affect CCRT in either males or females; live *L. kluyveri*, *P. kluyveri* and *P. nakasei* reduced female but not male CCRT, and dead yeasts had no effect on CCRT, with the exception of *P. nakasei*, which increased CCRT in both populations.

## DISCUSSION

The *Drosophila* gut microbiota is transient; flies acquire and replenish their gut microbes directly from their environment (Wong et al., 2013). The changing microbiota can modulate the host's response to environmental shifts (Fontaine et al., 2022). Although most studies have focused on the bacterial component of *D. melanogaster* microbiota, we show that yeasts are alive in the fly's digestive tract and improve host cold tolerance relative to axenic flies as much as the intact gut microbial complement.

The *D. melanogaster* gut bacterial microbiome shifts with the microbial composition of the food (Wong et al., 2013; but see Pais et al., 2018). Similarly, the community composition of yeasts in wild-caught adult *Drosophila* spp. is dependent on the food source from which they were collected (Chandler et al., 2012; Lachance et al., 1995). We found that males consumed almost no yeast, whereas females that were fed yeasts had engorged crops and abdomens (Fig. 3C; Fig. S1D). *Drosophila* females typically consume more food and feed more frequently than males (Wong et al., 2009), which is consistent with the pattern we observed. Although yeasts did not persist in the gut of males, yeast persistence in females depended on the yeast species (Fig. 4) – specifically, *S. cerevisiae*, which is not commonly associated with flies in nature, persisted for less time, and also had limited phenotypic impact relative to yeasts co-evolved with the *Drosophila* host.

We observed that not all yeast cells are digested: some survive after consumption and can be recovered throughout the gut of adult flies and in freshly deposited frass (Figs 2 and 3). Live yeasts have previously been reported from the guts of adult flies, and this survival has been attributed to the ability of spores to survive digestion (Coluccio et al., 2008; Reuter et al., 2007). However, after 24 h, *S. cerevisiae* was 60% less abundant in the gut than *L. kluyveri*, and although we found both vegetative cells and spores of *L. kluyveri* in the gut, we did not recover spores or live cells of *S. cerevisiae*, even though this strain does sporulate under laboratory conditions (M.-A. Lachance, pers. comm.). Thus, it appears that the *Drosophila*-derived species could relate to their host as symbionts, as well as sources of nutrients. Curiously, both these strains of yeast survived in the guts of *D. melanogaster* larvae, but *L. kluyveri* persisted longer than *S. cerevisiae* in the larval gut (Jiménez-Padilla et al., 2024). Hoang et al. (2015) also reported among-species variation in yeast persistence in *Drosophila* guts. Whether the host immune response drives these differences remains unknown (Stefanini, 2018), but reactive oxygen species in the gut reduce microbial load (Ha et al., 2009), raising the possibility that species-specific tolerance to oxidative damage may contribute to differential persistence among yeast species (Hoang et al., 2015).

We found that the gut microbiota modulates cold tolerance in female *D. melanogaster*: axenic flies had slower CCRT in females, but rearing axenic males did not change their CCRT (Fig. 4). This suggests that neither gut microbes nor their by-products influence male CCRT. A delay in chill coma recovery has been previously reported in microbiota-depleted *D. melanogaster* females, but males were not tested (Henry and Colinet, 2018). Contrary to our findings, Colinet and Renault (2014) found that live *S. cerevisiae* reduced CCRT in female flies in low-nutrition diets, suggesting a nutritional effect. In our study, feeding live *S. cerevisiae* to axenic flies did not change CCRT, and only yeast species isolated from wild *Drosophila* accelerated chill coma recovery (Figs 5 and 7). Thus, it appears that when the diet provides sufficient nutrients (our diet contains dead *S. cerevisiae*), the addition of live *S. cerevisiae* (or dead yeasts of any species) does not affect CCRT. However, interaction with live *L. kluyveri*, *P. kluyveri* and *P. nakasei* (even for just 48 h before cold exposure) speeds recovery from chill coma in a nutritionally independent fashion, such that females reared axenically recover as fast as conventionally reared flies.

Chill coma recovery time in *D. melanogaster* is plastic: cool rearing temperatures and acclimation to lower temperatures both reduce CCRT (Rako and Hoffmann, 2006; Ransberry et al., 2011), CCRT changes seasonally in nature (Behrman et al., 2015), and CCRT in tropical populations is slower than in temperate ones (Gibert et al., 2001; Hoffmann et al., 2002). The insect gut microbiota also varies with rearing temperature (Moghadam et al., 2018), geography (Lachance et al., 1995) and season (Ferguson et al., 2018). Does this rapidly changing gut microbiota contribute to the plasticity and adaptation of cold tolerance in the host? Increasing *L. kluyeri* abundance reduced CCRT (Fig. 6), suggesting some dose-dependence of the signal or activity of the yeast. Yeast species and abundance in *Drosophila* food sources (and guts) vary among populations (Lachance et al., 1995), which could contribute to geographic variation in CCRT. Similarly, if gut yeasts vary seasonally (as it does in bacteria; Ferguson et al., 2018), they could help determine seasonal patterns of CCRT. To our knowledge, there are no data linking yeast gut identity and abundance to individual CCRT in wild-caught flies to test the hypothesis that yeasts modulate cold tolerance in nature.

### How might live yeasts restore the CCRT phenotype in axenic adult female flies?

Any mechanism explaining the impacts of yeasts on adult CCRT needs to differ among yeast species, require activity by live yeast cells, act rapidly (within 48 h) and connect to the mechanisms of ion and water balance that determine CCRT (MacMillan et al., 2017). We identified three non-mutually exclusive potential mechanisms: gut yeasts could (1) modify (micro-) nutrient availability or digestive efficiency; (2) act through immune pathways that secondarily modify host physiology; or (3) directly modulate the physiology underlying recovery from chill coma.

Supplementing a low-nutrition diet with live *S. cerevisiae* increases lipid and protein content and modifies metabolic profiles of adult female *D. melanogaster* within 6 days (Colinet and Renault, 2014). Although nutritional status modulates cold tolerance in *D. melanogaster* (Colinet and Renault, 2014), diet manipulations do not reliably change cold tolerance traits (Colinet et al., 2013). We found that the macronutrient content of yeast cells does not speed CCRT, and that *S. cerevisiae* does not impact CCRT at all in our nutrient-replete diet. Thus, the faster CCRT we observed is probably not simply due to the improved macronutritional status of the flies. However, yeasts process food and produce a range of micronutrients, including B vitamins and unique sterols (such as ergosterol); these compounds are not essential but can have biological impacts (Dikkala et al., 2021; Starmer and Aberdeen, 1990). Such molecules would only be produced by living yeasts of the correct species, and could plausibly have a dose-dependent effect, as shown in Fig. 6. A single compound (or suite of compounds) that directly affects CCRT in the manner and magnitude we describe would be extremely novel and would likely shed light on the mechanisms underlying CCRT in general. Furthermore, the metabolic profiles of yeasts vary among species; in our experiment, a key difference between the genera *Lachancea* and *Saccharomyces* is that *Saccharomyces* ferments even when oxygen is available, whereas *Lachancea* is a facultative fermenter and likely produces less ethanol (Zhou et al., 2017).

Insect immune responses to fungi are mediated by receptors such as Gram-negative binding proteins (GNBPs) activating Toll signalling pathways, which have extensive phenotypic impacts beyond immunity (Roth, 2023). Cold exposure also initiates immune responses independently of infection in insects (El-Saadi et al., 2023), although the significance of this is not yet certain. Thus, there is capacity for yeasts to influence the (poorly known) pathways associated with plasticity in CCRT via cross-talk from immune pathways.

Gut yeasts may influence chill coma recovery by modulating fly physiology. When in chill coma, insects lose ion and water balance, and recovery requires active re-establishment of homeostasis, particularly clearing excess extracellular potassium and restoring membrane potential (Overgaard and MacMillan, 2017). The gut is a core component of iono-osmoregulation in insects (MacMillan et al., 2017), and gut function is influenced by the gut microbiota (Liu et al., 2022). Thus, we hypothesise that live yeasts could protect or restore gut integrity and function during cold exposure and recovery and thus affect CCRT. More prosaically, gut yeasts and their metabolites in the gut could alter gut osmolarity, directly affecting water and ion fluxes with the haemolymph (MacMillan et al., 2012). Indirectly, immune stimulation by yeasts in the gut could activate pathways related to Malpighian tubule physiology, increasing the fly's capacity to regain homeostasis after cold exposure (Davies et al., 2012).

### Ecological and evolutionary implications

We show here that yeasts enhance their host's cold tolerance in a sex- and yeast species-specific manner, even when microbe-free flies acquire the yeasts as adults. We observed the same pattern in two Australian populations that differed in intrinsic CCRT (Figs S4 and S5), suggesting that the yeast-mediated enhancement of female cold tolerance is a general effect across geographically and genetically distinct populations that differ in their innate cold tolerance (Lasne et al., 2018). Because *Drosophila* obtain their gut microbes from their environment, and larvae and adults often feed on different substrates, they may encounter different yeast species at each life stage. The transient nature of the gut microbiota means that flies can harbour different symbiotic partnerships over time, which may reverse or reinforce developmental plasticity in traits associated with responses to proximate thermal conditions. Psychrotolerant yeasts produce volatile compounds that attract *Drosophila* even in the cold (Liszkowska and Berlowska, 2021). We speculate that these yeasts may not only provide nourishment but also influence the fly's physiological responses to low temperatures.

### Conclusions

The relationship between *D. melanogaster* and their gut yeasts shapes host thermal tolerance. Rearing axenic female flies slows CCRT, but normal recovery times can be rapidly restored by reintroducing a single yeast species. This yeast-induced benefit is sex-specific, fast-acting, shows evidence of dose dependency, and appears restricted to yeast taxa more frequently associated with *Drosophila* in nature. Thus, gut microbiota could modulate cold tolerance independently of developmental conditions, potentially decoupling adult phenotypic plasticity from developmental history. Moreover, the magnitude of yeast-induced changes in CCRT is comparable to natural variation among *Drosophila* populations adapted to different thermal environments, suggesting that gut yeasts may be a significant source of phenotypic variation.

## Supplementary Material

10.1242/jexbio.251533_sup1 Supplementary information

## Data and resource availability

All data used in this study are available from a figshare repository at https://doi.org/10.6084/m9.figshare.29321777. All other relevant data and details of resources can be found within the article and its supplementary information.
