# Supplementary material for: Gut yeasts accelerate chill coma recovery in Drosophila melanogaster
Source: J Exp Biol. 2026 May 5;229(9):jeb251533. doi: 10.1242/jeb.251533 (PMC13200720; doi:10.1242/jeb.251533)
Supplement: Supplementary information [file jexbio-229-251533-s1.pdf]

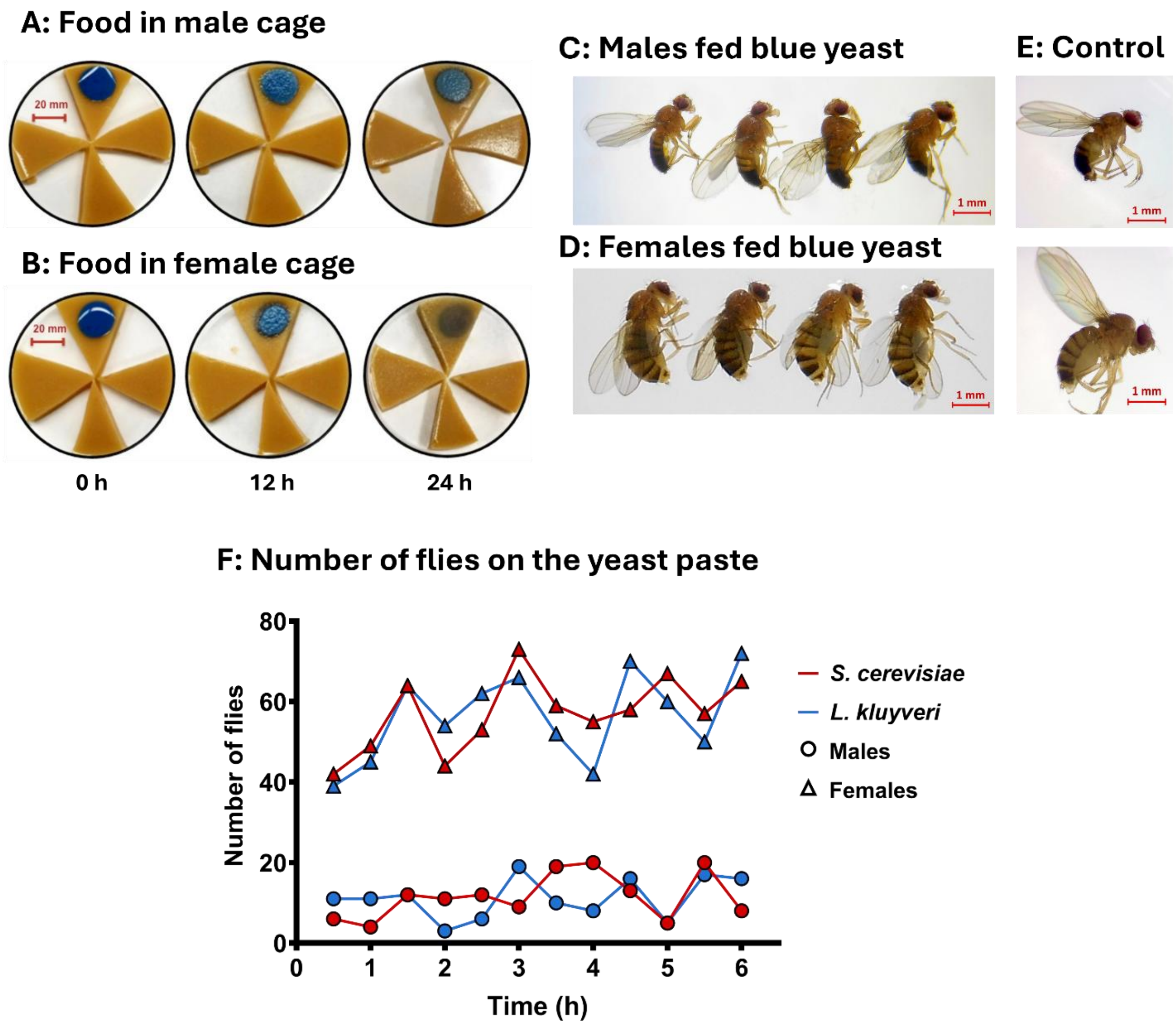

Fig. S1. Yeast consumption in male and female *D. melanogaster*. We anaesthetized axenic flies (48h post-eclosion) briefly with CO_2 to separate males and females into population cages (300 flies per cage). We fed the flies with a Petri dish with banana food topped with 50 µL yeast (*L. kluyveri* or *S. cerevisiae*) scraped from a culture plate and mixed with 50 µL of a 4 % Trypan blue solution (*A*, *B*). The cages were incubated under standard rearing conditions and monitored continuously until all the food in the female cage had been consumed (24 hours). Male flies (*C*) did not ingest the yeast paste as much as females did (*D*). Female's abdomens were enlarged with yeast, and the dark colour of the dye can be seen through their cuticle in contrast to control flies (*E*). We also recorded the number of flies aggregated at the yeast paste every 30 minutes (*F*). Female flies were more attracted to both yeast species paste than the males.

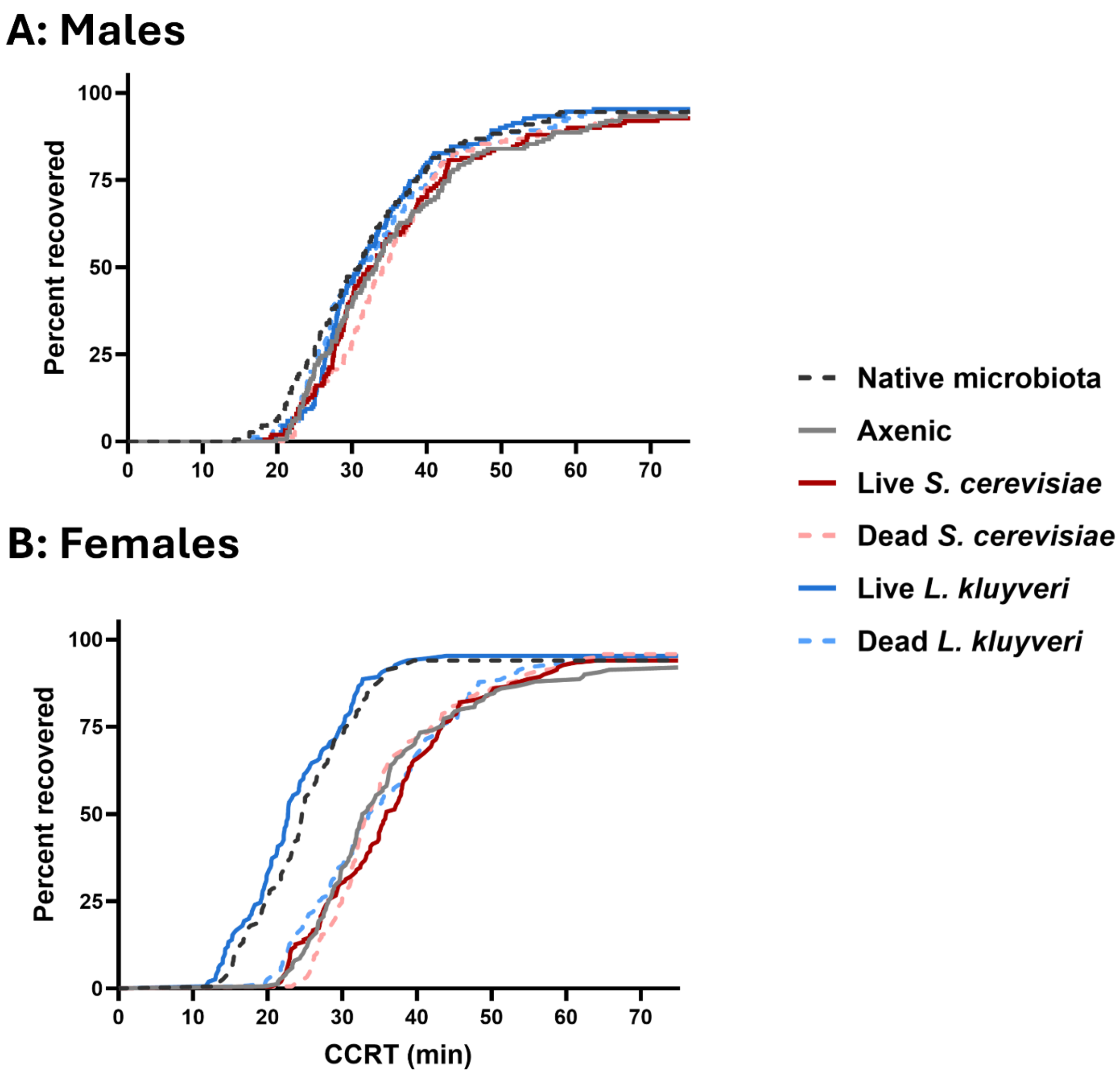

Fig. S2. Cumulative *D. melanogaster* chill coma recovery times. Data includes all recovery times for males (A) and females (B), with five flies per sex in each vial, including the 80% CCRT from the pooled data presented in Fig. 5 (n = 50 flies per treatment per sex; three cohorts). Cohorts did not differ within any treatment [Mantel-Cox Log-Rank test followed by a Benjamini-Hochberg False Discovery Rate (FDR) correction for multiple comparisons]. Statistics are provided in Table S1.

Journal of Experimental Biology • Supplementary information

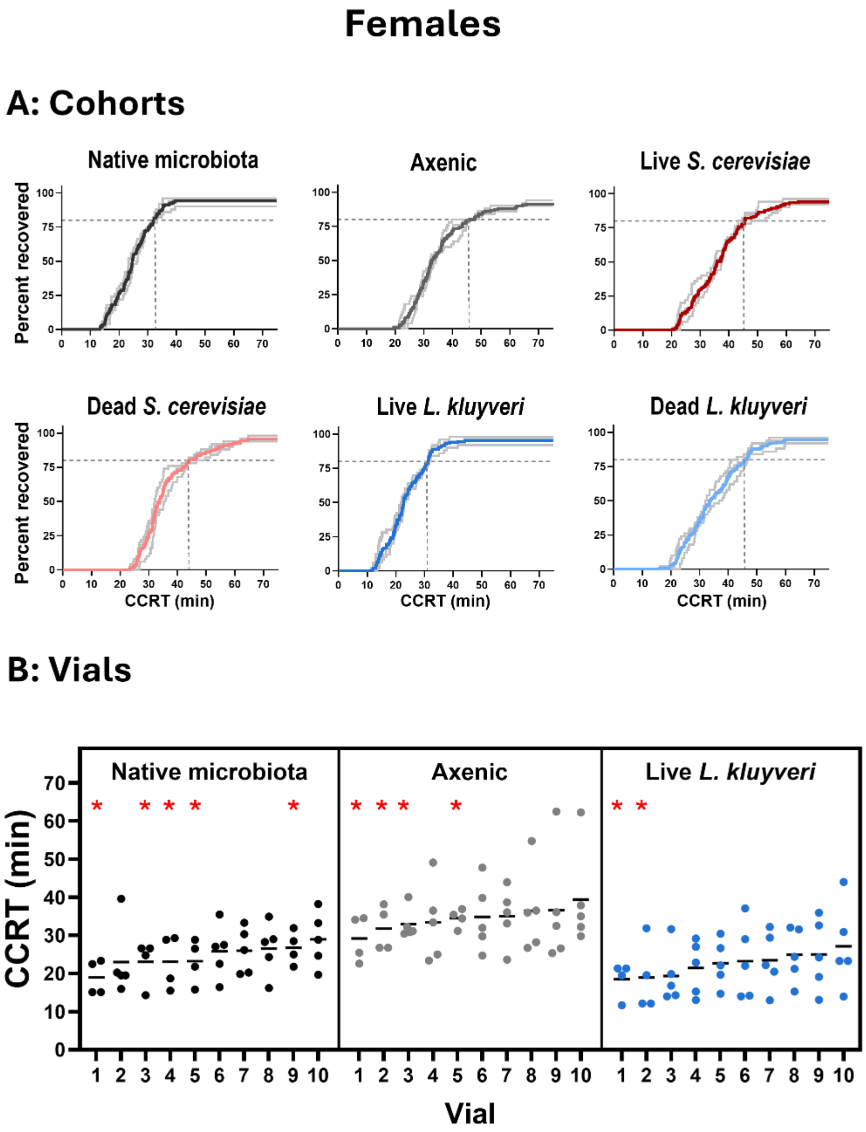

Fig. S3. Among-cohort and among-vial variation in *D. melanogaster* female chill coma recovery time. (A) Cumulative curves of CCRT from the data presented in Fig. S2B, separated by cohorts. Cohort curves are shown in grey, and coloured lines are the pooled data shown in Fig. S2B for comparison. Dashed horizontal lines indicate the time at which 80% of the flies had recovered, corresponding to the 80% (mean ± SD) CCRT values presented in the main manuscript (Fig. 5). Cohorts did not differ within any treatment [Mantel-Cox Log-Rank test followed by a Benjamini-Hochberg False Discovery Rate (FDR) correction for multiple comparisons]. Statistics are provided in Table S1. (B) Representative plots for three treatments showing individual CCRT values separated by vial to illustrate within-vial (inter-individual) variation; asterisks represent flies that did not recover during observation time. Data are from Cohort 1and part of the dataset presented in Fig. 5 and S2B and panel A. All data are available in the figshare repository.

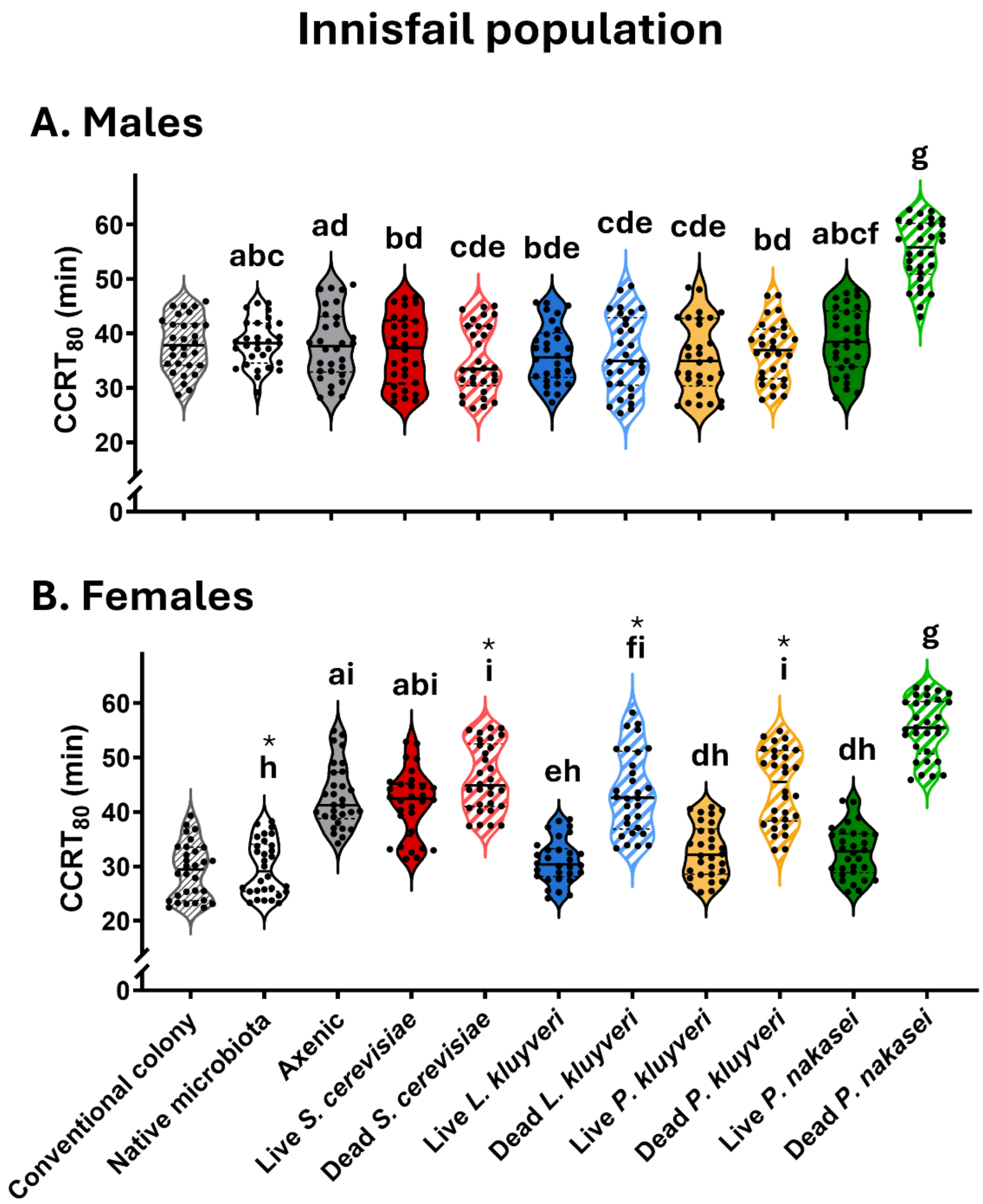

Fig. S4. **Effect of gut yeasts on the chill coma recovery time of adult *Drosophila melanogaster* collected from Innisfail, Queensland, Australia in 2016 by Carla Sgró.** The data are presented in two different panels for easy comparison of treatment within the same sex. $CCRT_{80}$ represents the time at which 80 % of the flies in a group of five had recovered. The effects of gut yeasts on both male (*A*) and female (*B*) flies were recorded as part of the same experiment and thus are analyzed together. The conventional colony group is included to demonstrate that there was no difference in CCRT compared to the native microbiota treatment, but it was not included in the overall analysis. Groups were compared using a 2-way ANOVA (treatment: $F_{9,580} = 69.63$, $P < 0.001$; sex: $F_{1,580} = 8.38$, $P < 0.01$; interaction: $F_{9,580} = 19.9$, $P < 0.001$), and significantly different groups by Tukey's HSD post-hoc test are indicated by different letters ($P < 0.05$). The asterisks above females indicate sex differences within the same treatment group. Each point represents the $CCRT_{80}$ from a group of five flies from a single vial (n = 30 vials per treatment, three cohorts pooled).

Journal of Experimental Biology • Supplementary information

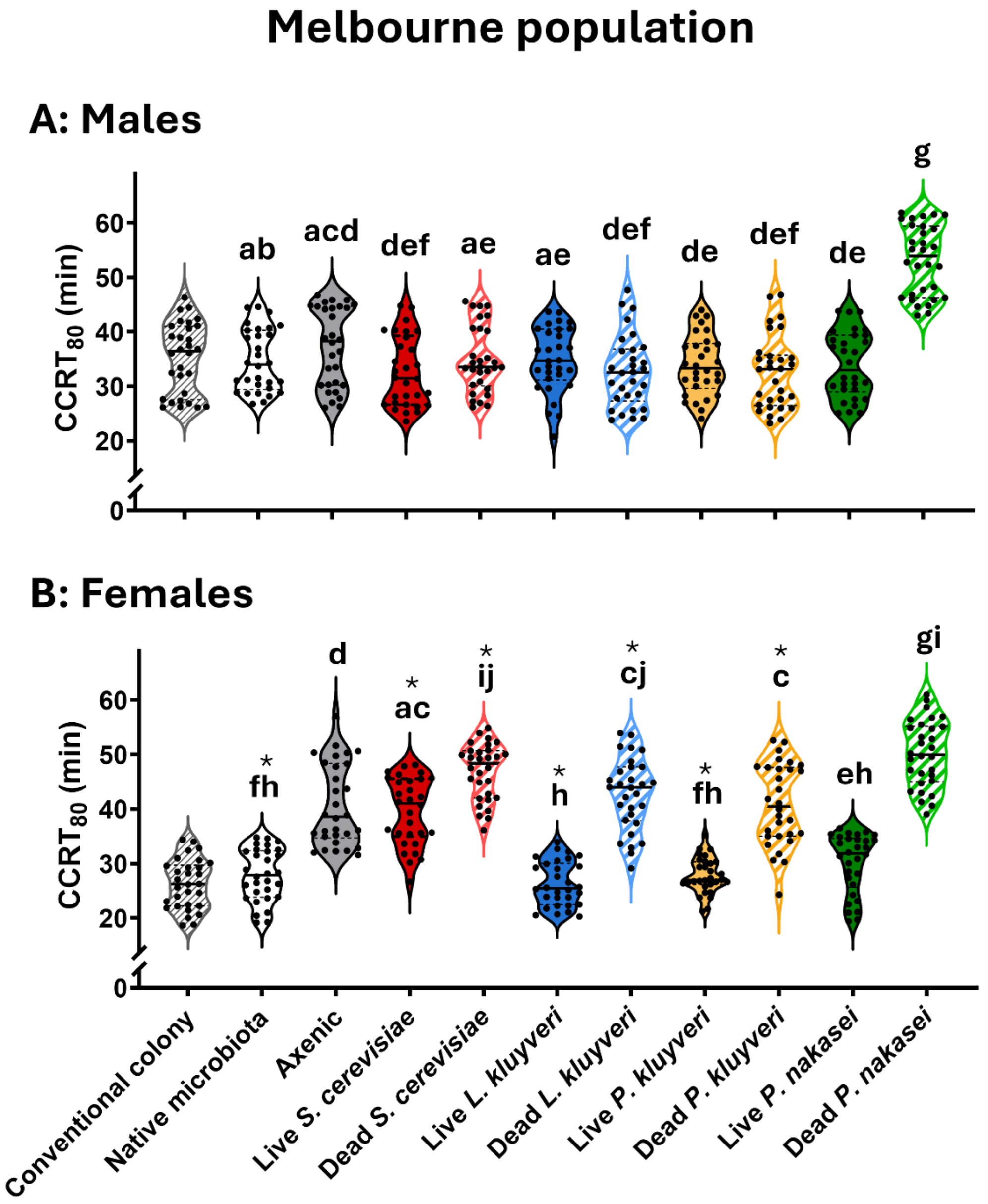

Fig. S5. Effect of gut yeasts on the chill coma recovery time of adult *Drosophila melanogaster* collected from Melbourne, Victoria, Australia in 2016 by Carla Sgró. The data are presented in two different panels for easy comparison of treatment within the same sex. $CCRT_{80}$ represents the time at which 80 % of the flies in a group of five had recovered. The effects of gut yeasts on both male (*A*) and female (*B*) flies were recorded as part of the same experiment and thus are analysed together. The conventional colony group is included to demonstrate that there was no difference in CCRT compared to the native microbiota treatment, but it was not included in the overall analysis. Groups were compared using a 2-way ANOVA (treatment: $F_{9,580} = 66.93$, $P < 0.001$; sex: $F_{1,580} = 6.03$, $P = 0.014$; interaction: $F_{9,580} = 24.29$, $P < 0.001$), and significantly different groups by Tukey's HSD post-hoc test are indicated by different letters ($P < 0.05$). The asterisks above females indicate sex differences within the same treatment group. Each point represents the $CCRT_{80}$ from a group of five flies from a single vial (n = 30 vials per treatment, three cohorts pooled).

Journal of Experimental Biology • Supplementary information

Table S1. Summary statistics for Figures S2 and S3. The Mantel-Cox curves include all data points per vial (Pooled data: 150 flies/treatment/sex; three cohorts of 50 flies each). Data complementary to the 80% CCRT presented in Figure 5. Table-wide FDR correction did not alter significance for cohort comparisons. Unadjusted values shown. Terms in bold typeface indicate statistical significance.

Data	Statistic	*P*
**Males (Fig. S2)**		
Pooled data	*df*=5, χ^2=9.92	0.078
Cohorts		
Native microbiota	*df*=2, χ^2=0.35	0.839
Axenic	*df*=2, χ^2=1.52	0.469
Live *S. cerevisiae*	*df*=2, χ^2=1.85	0.397
Dead *S. cerevisiae*	*df*=2, χ^2=0.93	0.629
Live *L. kluyveri*	*df*=2, χ^2=2.89	0.235
Dead *L. kluyveri*	*df*=2, χ^2=0.94	0.624
**Females (Fig. S3)**		
Pooled data	***df*=5, χ^2=165.8**	**<0.001**
Cohorts		
Native microbiota	*df*=2, χ^2=0.37	0.504
Axenic	*df*=2, χ^2=0.11	0.948
Live *S. cerevisiae*	*df*=2, χ^2=0.62	0.733
Dead *S. cerevisiae*	*df*=2, χ^2=2.42	0.298
Live *L. kluyveri*	*df*=2, χ^2=0.86	0.651
Dead *L. kluyveri*	*df*=2, χ^2=1.65	0.438

Journal of Experimental Biology • Supplementary information
